# Association between atherosclerosis and tooth loss in adult patients: systematic review and meta-analysis

**DOI:** 10.1038/s41432-026-01215-1

**Published:** 2026-03-18

**Authors:** Juan David Velasco-Ceballos, Juan Pablo Varela, Gloria Patricia Baena-Caldas, Martha Lucia Rodríguez, Johana Alejandra Moreno-Drada

**Affiliations:** 1 School of Dentistry, Universidad del Valle, Cali, Valle del Cauca Colombia; 2 Department of Pathology, SUNY Downstate Health Sciences University, New York, NY USA

**Keywords:** Occupational health, Dental conditions

## Abstract

**Objective:**

To evaluate the association between atherosclerosis and tooth loss in adults.

**Methods:**

This systematic review and meta-analysis addressed the PECO question: Is there an association between atherosclerosis (E) and tooth loss (O) in adults (P)? Observational studies conducted in individuals over 18 years old with atherosclerosis were considered. The literature search employed controlled and uncontrolled vocabulary in PubMed (MEDLINE), Scopus, Web of Science, and LILACS databases. Two reviewers independently assessed titles, abstracts, and full texts, followed by the extraction of relevant data. The information was narratively synthesized, and the methodological quality was assessed using the Joanna Briggs Institute (JBI) checklists. The researchers used the R v.4.5.1 software program for meta-analysis. The results were presented in forest plots, using mean differences and odds ratios with their respective 95% confidence intervals (95%CI) (PROSPERO CRD42025630179).

**Results:**

The search identified 390 records, from which, after removing duplicates and applying the inclusion criteria, 13 studies were selected for qualitative analysis and 12 for quantitative analysis. On average, individuals with atherosclerosis experienced significantly greater tooth loss, with 3.08 teeth lost (95%CI: 1.53;4.64), compared to those without the disease. However, the researchers observed no statistically significant association between atherosclerosis and the presence of severe tooth loss (OR 1.27 95%CI: 0.45;3.56). The methodological quality assessment revealed a low risk of bias.

**Discussion:**

Tooth loss was associated with atherosclerosis, potentially due to shared inflammatory, metabolic, and vascular mechanisms. Due to heterogeneity, confounding factors, and the low certainty of the evidence, the results should be interpreted cautiously. However, the findings underscore the importance of integrating oral health into cardiovascular risk assessments and support the systemic relevance of managing oral diseases in adults.

**Conclusions:**

The evidence confirmed an association between missing teeth and atherosclerosis. However, because of the included studies, this association cannot be interpreted as a clinical predictor or as a consequence of atherosclerosis. In this context, future research should examine the potential of tooth loss as an indicator that clusters with atherosclerotic burden and other related systemic factors.

Key Points

- Tooth loss is associated with atherosclerosis: The meta-analysis of 13 studies involving 6680 patients revealed that individuals with atherosclerosis experienced, on average, three more missing teeth than those not exposed to the disease. However, because of the included studies, this association cannot be interpreted as a clinical predictor or as a consequence of atherosclerosis.
- Atherosclerotic patients tend to present more systemic risk factors: The exposed group showed higher rates of smoking, hypertension, diabetes, and increased BMI, reinforcing the link between a pro-inflammatory systemic profile, oral disease, and vascular pathology.
- The importance of oral health and its connection to systemic health: dental professionals may play a crucial role in highlighting how oral health indicators contribute to public-health planning, early risk stratification, and the integration of oral and systemic health strategies.

## Introduction

Atherosclerosis is a chronic immunoinflammatory disease affecting medium and large arteries, caused by the accumulation of lipids in the vascular walls. Between 2000 and 2019, according to the World Health Organization (WHO), exposure to atherosclerosis triggered cardiovascular^1^ and cerebrovascular diseases, which represent the main cause of mortality worldwide^1,2^.

Atherosclerosis is attributed to several factors, including genetic and environmental^3^. Chronic inflammation is another key factor in the pathophysiological alterations that initiate and develop atherosclerosis^4^. The rupture of unstable atherosclerotic plaques, combined with platelet aggregation, thrombus formation^5^, and bacteremia^6^, could cause stenosis of blood vessels, triggering the clinical manifestations of acute cardiovascular diseases^7^. Some authors have identified the relationship between atherosclerosis, periodontal disease, and the role of inflammation in both^8–10^.

Periodontal disease is the leading cause of tooth loss^11^, but other infectious and inflammatory conditions in the mouth also play a significant role. These include dental caries, apical periodontitis, affected teeth caused by cysts or tumors, malocclusions, and trauma^12^. Each of these conditions can lead to the migration of bacteria from the oral cavity into the bloodstream^13–15^. Bacteria can adhere to the walls of the vessels, contributing to the formation of atherosclerotic plaque^6,16^. Among the infectious diseases with the greatest impact on human health, dental caries is the most prevalent globally, affecting an estimated 2.5 billion people, according to the WHO^17^.

Caries is a dynamic and multifactorial process that involves cycles of demineralization and remineralization, driven by bacterial metabolism within the dental structure. As the disease progresses, it causes a progressive loss of minerals, eventually leading to the formation of cavities and, ultimately, tooth loss^18^. Multiple microorganisms participate in the pathogenesis of caries, including Streptococcus mutans (S. mutans), Lactobacillus spp., and Actinomyces spp., being S. mutans the most relevant agent in this condition^19^. In several animal and clinical studies, S. mutans has been shown to induce atherosclerosis by harming vascular endothelial cells^20–23^. These microorganisms and their products can lead to leukocyte migration from the vascular system to the atherosclerotic endothelium and trigger the production of pro-inflammatory mediators, such as cytokines (IL-1β, TNF-α, IL-6, and IL-8) and inflammatory proteins. These mediators can stimulate the expression of adhesion molecules and their receptors in endothelial cells, leukocytes, macrophages, and neutrophils. As a result, migration and adhesion of these cells to the vascular tissues affected by atherosclerosis are promoted^24^.

Considering the different oral conditions that trigger tooth loss and involve inflammation or infection, currently, there is no synthesis of evidence regarding the impact of tooth loss as an oral health outcome related to atherosclerosis, aside from systematic reviews examining the link between periodontitis and atherosclerosis^25–27^. This work hypothesizes that a relationship exists between atherosclerosis and tooth loss due to various oral pathologies.

Therefore, this study aimed to explore the association between Atherosclerosis, as a systemic vascular condition, and tooth loss, as an indicator of oral health, in an adult population through a systematic review and meta-analysis of observational studies.

## Methods

This systematic review and meta-analysis followed the PECO strategy. The population (P) consisted of adult participants exposed (E) to atherosclerosis, who were compared with those unexposed (C) to atherosclerosis to observe the association with tooth loss (O). Highlighting that the exposed group consisted of participants with atherosclerosis, peripheral arterial disease (PAD), and carotid artery calcifications, considering that PAD and calcifications are indicators of atherosclerotic disease^28–31^. On the other hand, the unexposed group was defined as having a low atherosclerotic burden.

### Search strategy

The researchers searched electronic databases, including MEDLINE (PubMed), Scopus, Web of Science, and LILACS. To identify gray literature, including technical reports, theses, preprints, and conference proceedings, the researchers conducted a complementary search on Google and Google Scholar. The search strategy used controlled and uncontrolled vocabulary, considering the keywords “Adult”, “Atherosclerosis”, and “Tooth Loss” (Supplementary Table 1). There was no restriction on language or publication date. The search was conducted until March 2024. Articles published in languages other than English were assessed using machine translation tools (e.g., Google Translate). If any issues arose with the translated content, multilingual researchers or language experts were consulted. Duplicates of the references obtained were removed using Rayyan (web version, https://rayyan.ai). The researchers reviewed the reference lists of articles related to the study topic to find references not available in the databases they used. Additionally, they contacted the corresponding authors to get the needed materials or clarifications when the cited documents could not be found or when the information in them was incomplete.

### Eligibility criteria

The researchers included observational studies such as case-control, cohort, and cross-sectional studies. They also included studies that reported the mean number of missing teeth or the number of individuals with severe tooth loss, defined as the loss of 20 or more teeth. Studies that assessed the method of evaluating the degree of disease or atherosclerotic burden using indicators, indices, or diagnostic tools were included. Studies without complete outcome information, those that indicated a risk of atherosclerosis but not a diagnosis of the disease, as well as those that did not report outcomes for the unexposed group, were excluded, along with systematic reviews, meta-analyses, literature reviews, clinical trials, expert opinions, case reports, and editorials.

### Selection and extraction of data

Before selecting the studies, the reviewers were standardized by an expert, and the Kappa coefficient was obtained to corroborate reliability. Two independent reviewers blindly assessed the titles and abstracts, then the full texts to select studies that met the eligibility criteria using Rayyan (web version, https://rayyan.ai). The reviewers excluded citations that did not include the population of interest, identifying the outcome of tooth loss in the group exposed and, in the unexposed group, to atherosclerosis (Supplementary Table 2). An expert resolved the discrepancies between reviewers.

Two independent reviewers assessed the methodological quality of the studies in a blinded manner, and a third reviewer resolved any discrepancies. The Joanna Briggs Institute (JBI) checklists for cross-sectional, cohort, and case-control studies were used to determine the low, high, or unclear risk of bias.

Two reviewers extracted data from the included studies using a standardized format, and a third researcher resolved disagreements. They verified the accuracy of the extracted data twice. The reviewers collected the same variables for both the exposed and unexposed populations. The data included the year of publication, author, country, study design, number of participants, participant characteristics (such as age and sex), type of atherosclerosis, affected vessel, diagnostic tools, and outcomes: the number of missing teeth and the number of individuals with severe tooth loss (defined as loss of more than 20 teeth). They also analyzed potential confounding factors, including BMI, hypertension, smoking, and diabetes, along with the results of multivariate analyses from the included articles.

### Data synthesis

The researchers performed a narrative synthesis in tables and figures and a meta-analysis. Statistical heterogeneity was measured with the I² statistic, applying a random effects model due to the high heterogeneity observed. The meta-analysis results were expressed as a mean difference (MD) for the number of missing teeth and the Odds ratio (OR) for the number of individuals with severe tooth loss (loss of more than 20 teeth), along with 95% confidence intervals, using the R version 4.5.1 software program. The researchers used the meta and dmetar packages. To calculate the MD for the number of missing teeth, the researchers used a random-effects model, assuming heterogeneity among studies, and they employed the Restricted Maximum Likelihood (REML) method to estimate the variance between studies. They also adjusted the confidence intervals using the Hartung–Knapp method, given that the meta-analysis had a small number of studies and high heterogeneity. For the outcome of severe tooth loss, the researchers employed the Mantel–Haenszel (MH) method to calculate the OR using a random-effects model. To estimate the variance between studies, they used the Paule–Mandel (PM) method. Finally, they again adjusted the confidence intervals using the Hartung-Knapp method. The researchers performed a subgroup analysis considering studies exposed to atherosclerosis, those with peripheral arterial disease, and those with calcifications. For the meta-analysis of the number of missing teeth, the researchers performed a meta-regression using the Restricted Maximum Likelihood (REML) method, considering mean age as a moderator across the included studies, to examine the influence of age on the effect size. For the meta-analysis of severe tooth loss, the researchers did not perform a meta-regression, as the meta-analysis included only four studies, and the coefficient estimates could be unstable. Finally, the researchers performed a sensitivity analysis by excluding studies one by one to check the consistency of the results and a funnel plot to assess asymmetry and the risk of publication bias.

The researchers followed the Grading of Recommendations, Assessment, Development and Evaluation (GRADE) approach to rate the certainty of the evidence and produced a Summary of Findings using GRADEpro^32^. The certainty of the evidence was considered in relation to the risk of bias, inconsistency, indirectness, imprecision, or publication bias, and for each outcome.

The results of the meta-analysis are presented in forest plots. The protocol was registered in the International Prospective Register of Systematic Reviews (PROSPERO) (CRD42025630179).

### Ethics declarations

This study is based on previously published literature and did not involve human participants or animals; therefore, it does not require ethics committee approval. All data analyzed in this study are from previously published articles available in public databases.

## Results

**Fig. 1:**
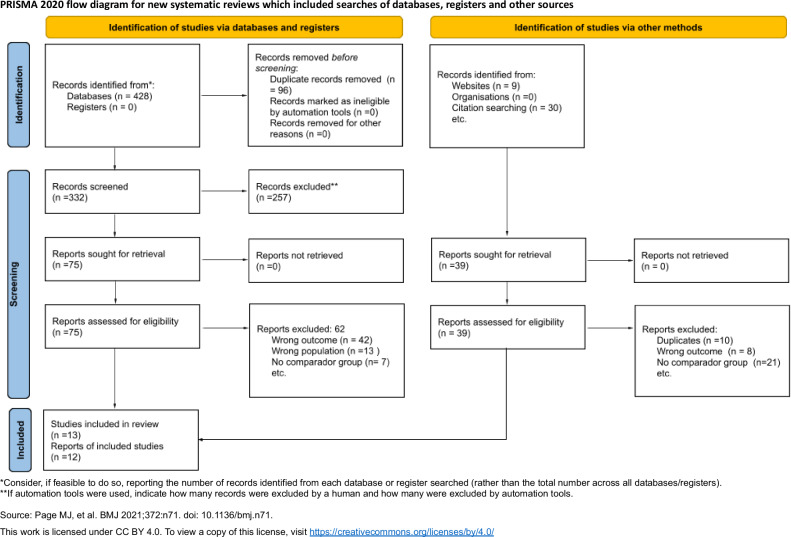
Flow Diagram.

The researchers found 390 articles through the database search and 39 through searches in external sources. After removing duplicates, they read the titles and abstracts of 307 records. Following the eligibility criteria, 72 articles were available for full-text reading, of which thirteen were included for qualitative analysis^28–31,33–41^ and 12 for quantitative analysis^28–31,33–35,37–41^ (Fig. 1). Of the 13 articles included in the qualitative analysis, 12 were cross sectional, and one was a case-control study. Studies were conducted in Brazil, the Netherlands, China, Turkey, the United States, Japan, Korea, Mexico, and India (Table 1).

**Table 1:** Characteristics of the included studies.

Author/ Year.	Country.	Study design	Population.	Group 1 (G1).	Group 2 (G2).	Outcome.	Outcome Measure. G1 / G2.	*P*
Gomes et al. 2012	Brazil	Cross -sectional	374 G1: 183 G2: 191 Male 241 Female 140	High CAB (FS > 7) by angiography	Low CAB (FS ≤ 7).	Patients with TL	G1: < 20 teeth 135 (73,8%) ≥ 20 teeth 48 (26,2%) G2: < 20 teeth 117 (61,3%). ≥ 20 teeth 74 (38,7%).	0.01.
Donders et al. 2020	Netherlands	Cross -sectional	212 G1: 142 G2: 70 Male 114 Female 98	CAC Tertile 2 y 3 by CT-scan	CAC tertile 1.	Tooth loss	G1: 10,5 ± 7,2 G2: 7,6 ± 6,6	0.033.
Soto-Barreras et al. 2013	Mexico	Case Control	60 G1: 30 G2: 30 Male 17 Female 43	PAD by ABI	No PAD	Tooth loss	G1: 13,6 ± 6,9^3–24^ G2: 9,8 ± 4,4^2–17^	0.0459
Donders et al. 2021	Netherlands	Cross -sectional	71 G1: 26 G2: 45 Male 31 Female 40	CAC ≤ 1 by CT-scan	CAC 0	Tooth loss	G1:5,5 ± 3,6 G2: 5,3 ± 3,2	0.177
				Framingham Risk Score CAC ≤ 1	Framingham Risk Score CAC 0		G1: 6,8 ± 5,9 G2: 2,9 ± 3,1	0.000
Bilgin Cetin et al. 2020	Turkey	Cross -sectional	309 G1: 233 G2: 76 Male 210 Female 99	CAD (+) by angiography	CAD (-)	Tooth loss	G1: 17^6–28^ G2: 9^3–24^	0.009
Sen S. et al. 2023*	USA	Cross -sectional	1145 G1: 344 G2: 801 Male 510 Female 635	<50% ICAS y ≥ 50% ICAS by MR angiogram	No ICAS	Patients with TL	G1:90 G2 166 *	N/D
Shen et al. 2023	China	Cross -sectional	272 G1: 88 G2: 184 Male 146 Female 126	Coronary Atherosclerosis Moderate-severe by CT-scan	Coronary Atherosclerosis Normal-Mild	Tooth loss	G1: 8,56 ± 7,54 G2: 4,99 ± 7,3	0.016
Ahn et al. 2016	Korea	Cross –sectional	1343 G1: 368 G1.1: 71 G2: 975 G2.1: 1271 Male: 842 Female: 501	Subclinical Atherosclerosis -cIMT by ultrasound	No Subclinical Atherosclerosis	Tooth loss	G1: 9,4 ± 7,9 G2: 6,3 ± 7,0	<0.0001
				PAD	No PAD		G1: 9,1 ± 8.0 G2: 7,0 ± 7,3	0.018
Thayana S et al. 2020	Brazil	Cross -sectional	418 Male: 219 Female: 199	CAB ≥ 50% by CT angiography	CAB < 50%	Patients with TL	G1: None/low (0–8 MT): 8 (10,8%). Mild (9–22 MT): 27 (36,5%). Severe (23–28 MT): 39 (52,7%) G2: None/low (0–8 MT): 81 (23,5%). Mild (9–22 MT): 137 (39,8%). Severe (23–28 MT): 126 (36,6%)	N/D
H. Yu 2014	China	Cross –sectional	847 G1: 245 G2: 602 Male: 434 Female: 413	cIMT>1,2 by ultrasound	cIMT<1,2	Tooth loss	G1: 4,62 ± 4,87 G2: 3,60 ± 4,34	0.004
Shimizu, Y, et al. 2022	Japan	Cross –sectional	1235 G1: 389 G2: 846 Male: 486 Female: 749	Presence of functional atherosclerosis -cIMT by ultrasound	Absence of functional atherosclerosis	Patients with TL	G1: 166 (43%) G2: 240 (28,3%)	N/D
Ahmed J et al. 2022	India	Cross -sectional	110 G1: 19 G2: 91 Male: 48 Female: 62	Presence of calcification by CBCT-scan	Absence of calcification.	Tooth loss	G1: 10.05 ± 7.083 G2: 3.76 ± 2.267	0.001.
Lazzari de Onofre et al. 2021	Brazil	Cross -sectional	284 G1: 179 G2: 105 Male: 105 Female: 179	Presence of calcification by CBCT-scan.	Absence of calcification.	Tooth loss	G1: 18,03 ± 10,048 G2: 14,16 ± 9,352	0.001.

*CAB* Carotid Atherosclerotic Burden, *CAC* Coronary Artery Calcification, *PAD* Peripheral Arterial Disease, *CAD* Coronary Artery Disease, *ICAS* Intracraneal Atheroscleroris, *cIMT* Carotid Intima Media Thikness. *TL* Tooth Loss, *MT* Missing Teeth. *ND* No Data, *CT-scan* Computed tomography, *CBCT-scan* Cone Beam computed tomography, *ABI* The Ankle Brachial Index.

The population evaluated consisted of 6680 patients (3062 women and 3629 men) with an average age of 65.6 ± 10.04 years. Regarding the method of assessing the degree of disease or atherosclerotic burden, one article used the Friesinger score^33^; four articles used the CAC score^34,35,38,39^; two used the Ankle-Brachial Index (ABI)^28,29^; two others, the carotid intima-media thickness (cIMT)^29,40^; one applied the cardio-ankle vascular index (CAVI)^32^, and another defined the exposed group by a reduction of more than 50% in the diameter of one or more epicardial arteries^36^. Sen et al. relied on the Warfarin-Aspirin Symptomatic Intracranial Disease Trial^37^, while two studies focused on imaging Analysis^30,31^.

Four articles evaluated coronary arteries^33,35,36,38^. Three articles evaluated the common carotid artery^29,40,41^, but only one^39^ evaluated the common carotid and its bifurcation into external and internal carotid arteries. Two articles evaluated the internal carotid artery^30,31^. Only one of the included articles studied the cerebral arteries (anterior, middle, and posterior)^37^, and one article evaluated peripheral arteries without specifying which^28^.

The exposed group had a Body Mass Index (BMI) of 26.2 ± 5, while the unexposed group had a BMI of 25.5 ± 4.9^20,25,26,28,31,32^. Similarly, Cetin et al.^36^ and Shen et al.^38^ reported a higher proportion of overweight participants in the exposed group compared to the unexposed group. On the other hand, the average number of people who presented arterial hypertension was 57.9% for the exposed group and 49.6% for the unexposed group^20,25–28,31^. Shimizu et al. indicated that their exposed group had a systolic pressure of 144 ± 19 mmHg and a diastolic pressure of 83 ± 12 mmHg. In contrast, the unexposed group had a systolic pressure of 135 ± 19 mmHg and a diastolic pressure of 82 ± 11 mmHg^32^. Regarding the percentage of smokers, in the atherosclerosis-exposed group, 29.3% were current smokers^29,31,34,35,38,40,41^, while 26.9% were ex-smokers^25,26,29^, and 30.2% had never smoked^29,31,34,35,38,40^. In patients unexposed to atherosclerosis, 22.2% were smokers^29,31,34,35,38,40,41^, 15.7% were ex-smokers^34,35,38^, and 37.6% were never smokers^29,31,34,35,38,40^. Soto-Barreras et al. recorded a higher number of smokers in the exposed group^28^. Similarly, Thayana S et al. reported a higher proportion of smokers and ex-smokers in the exposed group^39^. Ahn et al. reported that the highest percentage of never-smokers was in the unexposed group^29^. Bilgin et al. reported that the exposed group consumed approximately 15 cigarettes per day, while the unexposed group consumed 17^36^. Finally, when evaluating diabetes, 24% of patients in the exposed group had diabetes^20,23–28^, while 19% of patients in the unexposed group had diabetes. Tanyana S et al.^39^ and Anh et al.^29^ also reported that the percentage of diabetic patients in the exposed group was higher than that in the unexposed group. Shen et al. reported a longer duration of diabetes in years in the exposed group^38^, H. Yu et al. presented higher fasting blood glucose levels in patients in the exposed group^40^, and Shimizu Y et al. reported higher levels of glycated hemoglobin (HbA1c) in the exposed group^41^ (Supplementary Table 3).

### Number of missing teeth and severe tooth loss

**Fig. 2:**
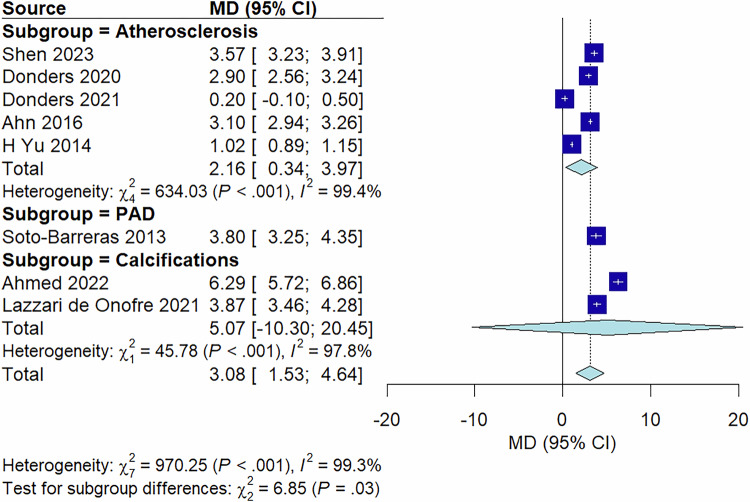
Forest plot. Tooth loss in individuals exposed and unexposed to atherosclerosis.

**Fig. 3:**
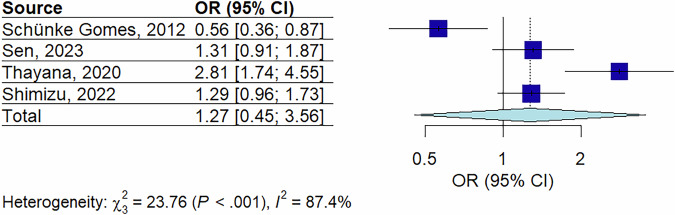
Forest Plot. Severe tooth loss in individuals exposed and unexposed to atherosclerosis.

Eight articles^28–31,34,35,38,40^ evaluated the total number of missing teeth expressed as mean and standard deviation, presenting a mean difference of 3.08 (95% CI 1.53; 4.64) (Fig. 2), indicating that the exposed group had a loss of approximately three more teeth than the unexposed group. Similarly, Bilgin Çetin et al. reported that the median tooth loss in the exposed group was 17^6–28^, while in the unexposed group, it was 9^3–24,36^. Four articles^33,37,39,41^ considered the number of patients who presented severe tooth loss in the exposed and unexposed groups. However, the results did not represent a statistically significant difference (OR 1.27 95% CI 0.45; 3.56) (Fig. 3).

The meta-regression analysis showed that age was not significantly associated with the effect size (β = –0.115; 95% CI –0.42 to 0.19; *p* = 0.38). The heterogeneity was high (I² = 99.1%), and the moderator explained 0% of the variability between studies. Similarly, the researchers conducted a meta-regression considering the age difference between the exposed and unexposed groups, also finding no significant association (β = 0.008; 95% CI: −0.63 to 0.65; *p* = 0.98; R² = 0.00%). Therefore, age does not appear to influence the effect in meta-analysis. However, the results should be interpreted with caution, given the limited number of studies and high heterogeneity.

Only two studies evaluated exposure and outcome by subgroup. Shen et al. found that in patients with short-term diabetes who presented moderate to severe atherosclerosis, a tooth loss of 8.62 (2.21) was observed, whereas in those with normal to average atherosclerosis, a tooth loss of 4.06 (0.8) was observed (*p* = 0.037). For patients with long-term diabetes who presented moderate to severe atherosclerosis, a tooth loss of 8.04 (1.35) was evident. On the other hand, those who presented normal to average atherosclerosis had a tooth loss of 6.57 (1.49) (*p* = 0.472). The young population presented a tooth loss of 8.80 (2.54) in those with severe to moderate atherosclerosis and 3.28 (0.84) in those with normal to average atherosclerosis (*p* = 0.012). Finally, the study identified a tooth loss of 8.03 (1.29) in older patients with severe to moderate atherosclerosis and 6.17 (1.23) (*p* = 0.306) in those with normal to average atherosclerosis^38^. On the other hand, H. Yu et al. classified the population into two subgroups: hyperglycemic and euglycemic. In hyperglycemic patients exposed to atherosclerosis (cIMT >1.2), there was a tooth loss of 4.72 (4.89), while in the unexposed group (cIMT <1.2), the tooth loss was 3.72 (4.45) (*p* = 0.029). In euglycemic patients exposed to atherosclerosis, the tooth loss was 4.43 (4.8), and in the unexposed group, 3.46 (4.22) (*p* = 0.108)^40^.

Twelve articles performed multivariate analysis using regression models to control confounding factors, most of which were adjusted for age, gender, smoking, hypertension, diabetes, and BMI^28,29,31,33–41^. Schunke Gomes et al. and Thayana S et al. implemented prevalence regression models^33,39^, whereas Bilgin Cetin et al., Sen S. et al., Shen et al., Ahn et al., and Shimizu Y et al. performed logistic regression analyses, identifying statistically significant differences between exposure and tooth loss^29,36–38,41^. The study by Ahmed J et al. did not perform multivariate regression models^30^, and the remaining five studies found no statistically significant differences.

Subgroup analysis considered articles that evaluated atherosclerosis itself, those that evaluated PAD, and those that assessed calcifications. Tooth loss was greater in individuals with atherosclerosis. The association between PAD and calcifications, with the number of missing teeth, requires further study. Only one article evaluated PAD, and the calcification subgroup showed no statistically significant association (two studies included), likely due to the small number of studies (Fig. 2). Similarly, the research team performed a subgroup analysis according to the diagnostic method for atherosclerosis; however, the results for each group showed no differences (Supplementary Figs. 3 and 4). Analysis by subgroups according to the different causes of tooth loss was not possible because the articles did not specify this information.

**Fig. 4:**
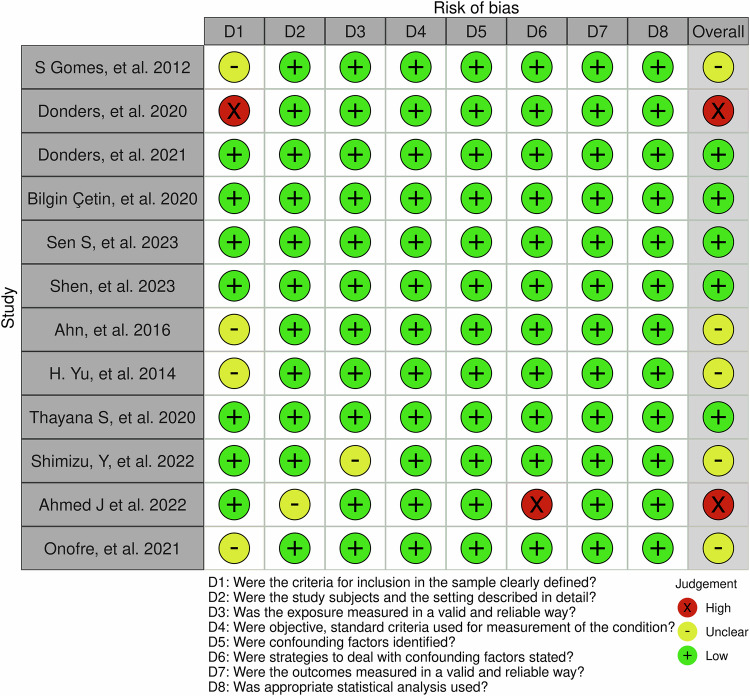
Risk of bias assessment Cross-sectional.

Regarding the assessment of publication bias, the funnel plot (Fig. 4) shows a dispersed data distribution, indicating there is no statistically significant evidence of publication bias. The researchers were unable to perform the Egger test to corroborate publication bias due to the small number of articles included (fewer than 10); therefore, the publication bias result should be interpreted with caution. The outcome of severe tooth loss included only four studies, so publication bias was not assessed. A sensitivity analysis was performed to ensure the robustness of the results, and the researchers found stability of the findings.

### Bias risk assessment

The researchers performed bias risk analysis using the JBI checklist in 12 cross-sectional studies and one case-control study. Schunke Gomez et al., Ahn et al., and Lazzari de Onofre et al. presented an uncertain risk of bias related to the eligibility criteria because the inclusion criteria were not clearly established in these studies^29,31,33^, while Donders et al. presented a high risk of bias for not providing such information^34^. In the description of the subject and their environment, only the article by Ahme J et al. presented an uncertain risk because it provided unclear information regarding the population group. In addition, it was the only study that presented a high risk of bias in the strategies to deal with confounding factors^30^. On the other hand, Shimizu et al. presented an unclear risk of bias regarding the reliability of the exposure measurement, as patients without functional atherosclerosis exhibited cIMT quartiles similar to those of patients with functional atherosclerosis^41^. Finally, all articles presented a low risk of bias when analyzing the criteria for measuring the condition, the presence of confounding factors, and the measurement of results (Fig. 4).

### Certainty of the evidence

The outcome regarding the number of missing teeth showed a very low level of certainty of evidence because the included studies were primarily cross-sectional and presented a serious risk of bias and indirectness. Similarly, the outcome regarding individuals with severe tooth loss also showed a very low level of certainty of evidence, as the included studies were cross-sectional, presented a serious risk of bias, indirectness, and exhibited very serious inconsistencies (Supplementary Table 4).

## Discussion

This study found an association between atherosclerosis and tooth loss in adults, consistent with previous research. Desvarieux et al. reported a positive association between carotid plaque prevalence and tooth loss in patients with periodontal disease, documenting that greater tooth loss was related to higher levels of atherosclerotic plaques^42^. Similarly, Chin et al. found that posterior tooth loss was associated with greater accumulation of atheroma in the arteries^43^.

While several studies support an association between periodontal disease and atherosclerosis^44–46^, Jung Y-S et al. found a link between tooth loss and subclinical atherosclerosis, but not with periodontal parameters^47^. This finding suggests that other mechanisms may be involved in explaining the link between tooth loss and atherosclerosis, which is consistent with the results in this study.

Several pathologies with an inflammatory component can cause tooth loss, including dental caries^48^. Glodny et al. suggested that dental caries could be an independent risk factor for atherosclerosis, comparable to periodontitis. Inflammatory spread from caries and apical periodontitis has been linked to increased atherosclerotic burden, while a higher number of dental fillings appears inversely related^49^. Along these lines, Ylöstalo PV et al. demonstrated a significant relationship between gingivitis, dental caries, and tooth loss with the onset of angina pectoris, supporting the role of oral conditions as potential cardiovascular risk indicators^50^.

In this review, the exposed group showed greater rates of elevated BMI, severe hypertension, smoking, and diabetes, consistent with existing literature. Diabetes and obesity share mechanisms such as insulin resistance and chronic inflammation, both contributing to atherosclerosis^51^. Obesity increases free fatty acids and promotes inflammation, while type 2 diabetes alters lipid metabolism and promotes fat accumulation, accelerating vascular damage and cardiometabolic risk^52^. Likewise, endothelial dysfunction, common in hypertension and atherosclerosis, alters the endothelium’s ability to regulate vascular tone, inflammation, and coagulation^53–55^. On the other hand, hypertension generates mechanical stress in the blood vessels, which induces the production of reactive oxygen species and increases oxidative stress^54^. This phenomenon exacerbates inflammation, weakens endothelial function, and accelerates the formation of atherosclerotic plaques^54,56^. Additionally, cigarette smoke generates reactive oxygen species that damage the endothelium and promote the formation of foam cells, a key step in atherogenesis^57^; these metabolic and environmental factors increase the risk of atherosclerotic disease.

This study has several limitations, as atherosclerosis is influenced by multiple confounding factors, including BMI, hypertension, smoking, and age^28,33,34,36,37,39–41,58,59^. Besides, other mechanisms, such as functional impairment, socioeconomic conditions, or access to oral care, can also contribute to tooth loss in patients with systemic diseases. Therefore, individuals with poorer overall health could lose teeth for reasons other than inflammation. However, seven articles employed multivariate models to control for confounding factors, mostly adjusting for age, gender, BMI, hypertension, diabetes, and smoking, and identified significant associations between tooth loss and atherosclerosis. Additionally, in the present study, the researchers performed a meta-regression to examine the potential influence of age on the effect; however, the meta-regression did not reveal any significant influence.

On the other hand, the lack of consistent adjustment for major confounding factors across studies increases heterogeneity and limits the comparability of results. Due to substantial heterogeneity in effect measures across studies, the researchers conducted a two-outcome analysis using a random-effects model to account for statistical heterogeneity.

Despite the association between periodontal disease and atherosclerosis reported in the literature^44–46^, this review did not find an association between severe tooth loss and atherosclerosis. This may be because only four articles were included. Additionally, since severe tooth loss is relatively common in some of the populations studied, the ORs may overestimate the strength of association relative to RRs; therefore, the interpretation of findings should be cautious. There is no statistically significant evidence of publication bias. The number of included studies also influences the outcome of the publication bias assessment. Although publication bias is not suspected, the minimum number of studies required to analyze publication bias accurately is 10, and in this study, each meta-analysis included fewer than 10. Furthermore, most of the studies included in the review were cross-sectional, a study design that does not allow for establishing causality. In fact, the studies did not report the reasons for tooth loss. Additionally, both outcomes showed very low certainty of evidence; therefore, results should be interpreted with caution. The researchers emphasize the need for studies with designs that can assess causality, using robust methods that ensure consistency, control for confounding factors, and minimize bias to produce accurate interpretations.

Despite its limitations, this review suggests that localized odontogenic factors may influence the association between tooth loss and atherosclerosis, highlighting the complex relationship between oral and systemic health, particularly in terms of cardiovascular and cerebrovascular risk. Furthermore, the methodological quality assessment identified only two studies at risk of bias, and over 45% of the studies were assessed as having acceptable methodological quality (low risk). Additionally, the researchers applied exclusion criteria that allowed them to analyze outcomes in both exposed and unexposed patients to obtain direct comparisons and adequately assess the association between exposure and the outcome of interest, with a comparator group serving as a reference.

## Conclusion

The association between tooth loss and atherosclerosis may reflect shared risk factors. The studies included in this review do not allow causal inference. Therefore, future research should examine whether tooth loss serves as an indicator associated with atherosclerotic burden and other related systemic factors, highlighting the importance of oral health and its connection to systemic health.

### Supplementary information

This document outlines the database search strategies (Supplementary Table 1), citations excluded (Supplementary Table 2), describes population characteristics including BMI, hypertension, and diabetes (Supplementary Table 3), shows the assessment of the certainty of the evidence (Supplementary Table 4), reports the Moose checklist, PRISMA abstract checklist, and PRISMA checklist (Supplementary Table 5, 6 and 7), presents the funnel plot used to assess publication bias (Supplementary Fig. 1), details the risk of bias assessment for the case-control study (Supplementary Fig. 2) and presents the forest plots of tooth loss and severe tooth loss in exposed and unexposed individuals according to diagnostic tools (Supplementary Fig. 3 and 4).

## Supplementary information

Supplementary Table 1. Search Strategy

Supplementary Table 2. Citations excluded.

Supplementary Table 3. Characteristics of the population

Supplementary Table 4. Certainty of evidence.

Supplementary Table 5. Meta-analyses Of Observational Studies in Epidemiology (Moose) Cheklist

Supplementary Table 6. Preferred Reporting Items for Systematic Reviews and Meta-analysis (PRISMA) 2020 for Abstracts Checklist

Supplementary Table 7. Preferred Reporting Items for Systematic Reviews and Meta-analysis (PRISMA) 2020 Checklist

Supplementary Fig. 1. Funnel plot Tooth loss in patients exposed and unexposed to atherosclerosis

Supplementary Fig. 2. Risk of bias assessment Case control

Supplementary Fig. 3. Forest Plot of tooth loss in patients exposed and unexposed to atherosclerosis according to the diagnostic tools.

Supplementary Fig. 4. Forest Plot of severe tooth loss in patients exposed and unexposed to atherosclerosis according to the diagnostic tools.

Supplementary information

## Data Availability

The data supporting the findings of this study, including the strategy search, list of included articles, data extraction tables, and quality assessment matrices, are publicly available in Mendeley Data at: Moreno Drada, Johana; Velasco Ceballos, Juan David; Varela Chicue, Juan Pablo; Rodriguez Paz, Martha Lucia; Baena-Caldas, Gloria Patricia (2026), “Tooth loss and Atherosclerosis”, Mendeley Data, V2, 10.17632/rhjbnddm5p.2.
