# Supplementary material for: Association between atherosclerosis and tooth loss in adult patients: systematic review and meta-analysis
Source: Evid Based Dent. 2026 Mar 18;27(2):42–3. doi: 10.1038/s41432-026-01215-1 (PMC13309286; doi:10.1038/s41432-026-01215-1)
Supplement: Supplementary file 1 — Supplementary Table 1. Search Strategy [file 41432_2026_1215_MOESM1_ESM.pdf]

Search strategy

(November 2025).

Search	Query	Records
Pubmed	((((((((Aged, 80 and over[MeSH Terms])) OR (Aged[MeSH Terms])) OR (Middle age[MeSH Terms])) OR (Adult[MeSH Terms])) OR (Young adult[MeSH Terms])) OR (Adults[Title/Abstract])) AND (((((((Atherosclerosis[MeSH Terms]) OR (Arteriosclerosis[MeSH Terms])) OR (Peripheral Arterial Disease[MeSH Terms])) OR (Arteriosclerosis Obliterans[MeSH Terms])) OR (Arterial Occlusive Diseases[MeSH Terms])) OR (Atheroscleroses[Title/Abstract])) OR ((((Large vessel disease[Title/Abstract]) OR (Large vessel aterosclerosis [Title/Abstract])) OR (Large artery aterosclerosis [Title/Abstract])) OR (Large artery atherosclerotic occlusive disease[Title/Abstract])))) AND (((Tooth Loss[MeSH Terms]) OR (Dental loss[Title/Abstract])) OR (Teeth loss[Title/Abstract]))	32
LILACS	((mh:(Adulto)) OR (mh:(Anciano)) OR (mh:(Anciano de 80 o más Años)) OR (mh:(Persona de Mediana Edad)) OR (mh:(Adulto Joven)) OR (Adultos) OR (Personas de Edad) OR (Personas Mayores) OR (Persona de Edad) OR (Ancianos) OR (Adulto Mayor) OR (Anciano de 80 Años o más) OR (Mediana Edad) OR (Personas de Mediana Edad) OR (Adulto de Mediana Edad) OR (Jóvenes Adultos) OR (Joven Adulto) OR (Adultos Jóvenes)) AND ((mh:(Aterosclerosis)) OR (mh:(Arteriosclerosis)) OR (mh:(Enfermedad Arterial Periférica)) OR (mh:(Arteriosclerosis Obliterante)) OR (mh:(Arteriopatías Oclusivas)) OR (Ateroesclerosis) OR (Arterioesclerosis) OR (Arteriopatía Periférica) OR (Arteriopatías Obstructivas) OR (Oclusión Arterial) OR (Enfermedad de grandes vasos) OR (Aterosclerosis de grandes vasos) OR (Aterosclerosis de grandes arterias) OR (Enfermedad oclusiva aterosclerótica de grandes arterias)) AND ((Pérdida de Diente) OR (mh:(Pérdida de Diente)) OR (Diente perdido))	23
Scopus	TITLE-ABS-KEY (aged) OR TITLE-ABS-KEY ("aged, 80 over") OR TITLE-ABS-KEY ("middle age") OR TITLE-ABS-KEY (adult) OR TITLE-ABS-KEY ("young adult") OR TITLE-ABS-KEY (adults) AND TITLE-ABS-KEY (atherosclerosis) OR TITLE-ABS-KEY (arteriosclerosis) OR TITLE-ABS-KEY ("peripheral arterial disease") OR TITLE-ABS-KEY ("arteriosclerosis obliterans") OR TITLE-ABS-KEY ("arterial occlusive diseases") OR TITLE-ABS-KEY (atheroscleroses) OR TITLE-ABS-KEY ("large vessel disease") OR TITLE-ABS-KEY ("large vessel aterosclerosis") OR TITLE-ABS-KEY ("large artery aterosclerosis") OR TITLE-ABS-KEY ("large artery atherosclerotic occlusive disease") AND TITLE-ABS-KEY ("Tooth Loss") OR TITLE-ABS-KEY ("loss, thooth") OR TITLE-ABS-KEY ("Dental loss") OR TITLE-ABS-KEY ("Teeth loss")	93
Web of Science	(TS=(Adult) OR TS=(Aged) OR TS=(Aged, 80 and over) OR TS=(Middle age) OR TS=(Young adult) OR ALL=(Adults)) AND (TS=(Atherosclerosis) OR TS=(Arteriosclerosis) OR TS=(Peripheral Arterial Disease) OR TS=(Arteriosclerosis Obliterans) OR TS=(Arterial	280

	Occlusive Diseases) OR ALL=(Large vessel disease) OR ALL=(Atheroscleroses) OR ALL=(Large vessel aterosclerosis) OR ALL=(Large artery aterosclerosis) OR ALL=(Large artery atherosclerotic occlusive disease)) AND (TS=(Tooth Loss) OR ALL=(Loss, Thooth) OR ALL=(Dental loss) OR ALL=(Teeth loss))	
