# Supplementary material for: Association between atherosclerosis and tooth loss in adult patients: systematic review and meta-analysis
Source: Evid Based Dent. 2026 Mar 18;27(2):42–3. doi: 10.1038/s41432-026-01215-1 (PMC13309286; doi:10.1038/s41432-026-01215-1)
Supplement: Supplementary file 2 — Supplementary Table 2. Citations excluded. [file 41432_2026_1215_MOESM2_ESM.pdf]

Supplementary Table 2. Citations excluded

| Author/ Year. | Journal. | Paper name | Exclusion criteria. |
|---|---|---|---|
| Desvarieux et al. 2003 | Stroke | Relationship between periodontal disease, tooth loss, and carotid artery plaque: The oral infections and vascular disease epidemiology study (INVEST). | There is no unexposed group and therefore no outcome for the unexposed group. |
| Desvarieux et al. 2004 | Stroke | Gender differences in the relationship between periodontal disease, tooth loss, and atherosclerosis. | There is no unexposed group and therefore no outcome for the unexposed group. |
| Hung et al. 2003. | Circulation | Oral health and peripheral arterial disease | There is no unexposed group; it evaluates the relative risk (RR) of developing PAD depending on the number of missing teeth. |
| Chin et al. 2010, | Journal of Advanced Prosthodontics | Relationship between tooth loss and carotid intima-media thickness in Korean adults | No unexposed group is presented; a regression analysis is presented to evaluate the effect of tooth loss on intima-media thickness. |

Supplementary Table 2. Citations excluded

| | | | |
|---|---|---|---|
| Asai, et al 2015 | JDR Clinical Research Supplement | Tooth loss and atherosclerosis: The Nagahama study | There is no unexposed group and therefore no outcome for the unexposed group. |
| Kurushima, et al 2015 | Plos One | Examination of the Relationship between Oral Health and Arterial Sclerosis without Genetic Confounding through the Study of Older Japanese Twins | There is no unexposed group and therefore no outcome for the unexposed group. |
| Del Brutto, O, et al. 2020 | Revista Ecuatoriana de Neurología | Intracranial atherosclerotic disease and severe tooth loss in community-dwelling older adults | There is no unexposed group and therefore no outcome for the unexposed group. |
| Jung Y.S, et al. 2013 | Journal of clinical periodontolo y | Relationship between periodontal disease and subclinical atherosclerosis: The Dong-gu study | There is no unexposed group and therefore no outcome for the unexposed group. |
| Holmlund & Lind 2012 | NA | Number of Teeth Is Related to Atherosclerotic Plaque in the Carotid Arteries in an Elderly Population | There is no exposed or unexposed group and therefore no outcomes. |

Supplementary Table 2. Citations excluded

| | | | |
|---|---|---|---|
| Pham et al. 2024 | Scientific reports | Impact of tooth loss and patient characteristics on coronary artery calcium score classification and prediction | There is no results for tooth loss, It was used as a mesurment for CAC |
| Song et al. 2025 | Plos one | The link between periodontitis and atherosclerotic cardiovascular disease in non Hispanic White adults: NHANES 1999 to 2014 | There is no clear atherosclerosis diagnosis |
| Dougham et al. 2025 | Journal of periodontal research. | Association of endogenous sex hormone levels with tooth loss due to periodontitis in men and post-menopausal women: The multi-ethnic study of atherosclerosis | There is no clear atherosclerosis diagnosis and there is no exposed group. |
| Soronzonbold et al 2024 | Journal of periodontal and implant science. | Measurement of atherosclerosis markers in individuals with periodontitis | There is no exposed or unexposed group and therefore no outcomes. |
| Takami et al. 2025 | Scientific reports | Longitudinal relationship between atherosclerosis and progression of periodontitis in community dwelling people in Nagasaki Islands Study | There is no exposed or unexposed group and therefore no outcomes. |

Supplementary Table 2. Citations excluded

| | | | | |
|---|---|---|---|---|
| . | Benjamin et al. 2024 | Gerontogical society of America | Poor Oral Health Is Associated With Inflammation, Aortic Valve Calcification, and Brain Volume Among Forager-Farmers | There is no results for tooth loss |
| | Swilem et al. 2024 | Cureus | The Impact of Periodontal Inflammation on the Severity of Coronary Atherosclerosis | There is no results for tooth loss |

**References:**


1. Desvarieux M, Demmer RT, Rundek T, Boden-Albala B, Jacobs DR, Papapanou PN, et al. Relationship between periodontal disease, tooth loss, and carotid artery plaque: the Oral Infections and Vascular Disease Epidemiology Study (INVEST). Stroke. septiembre de 2003;34(9):2120-5.

2. Desvarieux M, Schwahn C, Völzke H, Demmer RT, Lüdemann J, Kessler C, et al. Gender differences in the relationship between periodontal disease, tooth loss, and atherosclerosis. Stroke. septiembre de 2004;35(9):2029-35.

3. Hung HC, Willett W, Merchant A, Rosner BA, Ascherio A, Joshipura KJ. Oral health and peripheral arterial disease. Circulation. 4 de marzo de 2003;107(8):1152-7.

4. Chin UJ, Ji S, Lee SY, Ryu JJ, Lee JB, Shin C, et al. Relationship between tooth loss and carotid intima-media thickness in Korean adults. J Adv Prosthodont. diciembre de 2010;2(4):122-7.

5. Asai K, Yamori M, Yamazaki T, Yamaguchi A, Takahashi K, Sekine A, et al. Tooth Loss and Atherosclerosis. J Dent Res. marzo de 2015;94(3 Suppl):52S-58S.

6. Kurushima Y, Ikebe K, Matsuda K ichi, Enoki K, Ogata S, Yamashita M, et al. Examination of the Relationship between Oral Health and Arterial Sclerosis without Genetic Confounding through the Study of Older Japanese Twins. PLoS One. 26 de mayo de 2015;10(5):e0127642.

7. Del Brutto OH, Mera RM, Del Brutto VJ, Pérez P, Recalde BY, Costa AF, et al. Intracranial atherosclerotic disease and severe tooth loss in community-dwelling older adults. Revista Ecuatoriana de Neurologia. 2020;29(2):39-45.

8. Jung Y, Shin M, Kim I, Kweon S, Lee Y, Kim O, et al. Relationship between periodontal disease and subclinical atherosclerosis: The Dong-gu study. JOURNAL OF CLINICAL PERIODONTOLOGY. marzo de 2014;41(3):262-8.

9. Holmlund A, Lind L. Number of Teeth Is Related to Atherosclerotic Plaque in the Carotid Arteries in an Elderly Population. JOURNAL OF PERIODONTOLOGY. marzo de 2012;83(3):287-91.

10. Pham, T. D., Zou, L., Patel, M., Holmes, S. B., & Coulthard, P. (2024). Impact of tooth loss and patient characteristics on coronary artery calcium score classification and prediction. *Scientific Reports*, *14*, 28315.

11. Song, Q., Zhang, H., Su, Y., & Song, J. (2025). The link between periodontitis and atherosclerotic cardiovascular disease in non-Hispanic White adults: NHANES 1999 to 2014. *PLOS ONE*, *20*(4), e0321220.

Supplementary Table 2. Citations excluded

12. Doughan, M., Chehab, O., Doughan, B., Lima, J. A. C., & Michos, E. D. (2025). Association of endogenous sex hormone levels with tooth loss due to periodontitis in men and post-menopausal women: The multi-ethnic study of atherosclerosis. *Journal of Periodontal Research, 60*(1), 166–176.

13. Soronzonbold, A., Munkhkherlen, E., Batchuluun, K., Puntsag, O.-E., Shuumarjav, U., Batbayar, B., *et al.* (2024). Measurement of atherosclerosis markers in individuals with periodontitis. *Journal of Periodontal & Implant Science, 54*(1), 37–43.

14. Tamaki, N., Fukui, M., Kitamura, M., Fukuda, H., Furugen, R., Yamanashi, H., Miyata, J., Saito, T., & Maeda, T. (2025). Longitudinal relationship between atherosclerosis and progression of periodontitis in community-dwelling people in Nagasaki Islands Study. *Scientific Reports, 15*, 13437.

15. Trumble, B. C., Schwartz, M., Ozga, A. T., Schwartz, G. T., Stojanowski, C. M., Jenkins, C. L., Kraft, T. S., Garcia, A. R., Cummings, D. K., Hooper, P. L., Rodriguez, D. E., Buetow, K., Beheim, B., Irimia, A., Thomas, G. S., Thompson, R. C., Gatz, M., Stieglitz, J., Finch, C. E., Gurven, M., Kaplan, H., & HORUS Team. (2024). Poor oral health is associated with inflammation, aortic valve calcification, and brain volume among forager-farmers. *The Journals of Gerontology, Series A: Biological Sciences and Medical Sciences, 79*(5), 1–8.

16. Swilem, E. S., Elkenany, N. M., Algazzar, A. S., Youssef, N., Swilem, S. S., Gendia, E. A., Swillem, A. S., Elmalah, A. A., Sabah, Z., & Rasool, T. (2024). The impact of periodontal inflammation on the severity of coronary atherosclerosis. *Cureus*, *16*(4), e57653.

Supplementary Table 2. Citations excluded

Supplementary Table 2. Citations excluded
