# Supplementary material for: Association between atherosclerosis and tooth loss in adult patients: systematic review and meta-analysis
Source: Evid Based Dent. 2026 Mar 18;27(2):42–3. doi: 10.1038/s41432-026-01215-1 (PMC13309286; doi:10.1038/s41432-026-01215-1)
Supplement: Supplementary file 3 — Supplementary Table 3. Characteristics of the population [file 41432_2026_1215_MOESM3_ESM.pdf]

Supplementary Table 3. Characteristics of the population

Author/ Year.	Country.	BMI	Hypertension.	Smokers	Diabetes	Type of atherosclerosis.	Affected blood vessels
Gomes et al. 2012	Brazil	Normal = 81 (21,2%). Overweight = 152 (39,8%). Obesity = 104 (27,2%).	Without hypertension = 87 (22,9%). With hypertension= 293 (77,1%)	Non-smoker = 174 (45,6%) Smoker/ex-smoker = 205 (53,8%)	G1= Diabetes: 65(34,8%) No diabetes: 122(65,2%) G2= Diabetes: 39 (20,3%) No diabetes: 153 (79,7%)	Carotid atherosclerotic burden (CAB).	Coronary arteries
Donders et al. 2020	Netherlands	Total = 28,0 ± 4.9 (kg/m2). G1 = 28 ± 5,1 G2 = 28 ± 5.	Total = 128 (60%). G1 = 94 (73,4) G2 = 34 (26,6%)	Total = Current: 39 (18,4%). Former smoker: 86 (40,6%). Never: 86 (40,6%). G1= Current: 22 (15,4%). Former smoker: 65 (45,7%). Never: 54 (38%). G2= Current: 17 (43,6%). Former smoker: 21 (24,4%). Never: 32 (37,2%).	Total = 32 (15,1%) G1= 23 (16,2%). G2 = 9 (28,1%).	Atherosclerotic cardiovascular disease (ACVD).	Complete coronary tree
Soto-Barreras et al. 2013	Mexico	G1= 29,19 ±4,53 kg/m 2. G2= 27,96 ± 4,16 kg/m 2.	G1 N= 24 (80%). G2 N= 18 (60%).	G1 N= 15 (50%). G2 N= 10 (33%)	G1= 9 (30%) G2= 9 (30%)	Peripheral arterial disease (PAD)	Peripheral arteries.

Supplementary Table 3. Characteristics of the population

Donders et al. 2021	Netherlands	Total= 23,4 ± 6,0<br>G1= 24.6 ± 3.0<br>G2= 22.7 ± 7.2	Total= 20 (28,2%).<br>G1= 8 (30,8%)<br>G2= 12 (26,6%).	Total = Never smoked: 36 (51,4%)<br>Smoked: 34 (48,6%)<br>Smokes: 9 (12,7%)<br>G1 = Never smoked: 11 (44%)<br>Smoked: 14 (56%)<br>Smokes: 4 (15,4%)<br>G2 = Never smoked: 25 (55,6%)<br>Smoked: 20 (44,4%)<br>Smokes: 5 (11,1%)	G1= 0 (0,0%)<br>G2= 3 (6,7%)	Coronary atherosclerosis. (Coronary artery calcification).	Coronary artery.
Bilgin Cetin et al. 2020	Turkey	≥25 kg/m2 (Overweight):<br>G1= 189 (81,1%)<br>G2= 67 (88,2%)	G1= 161 (69,1%)<br>G2= 50 (65,8%)	Cigarettes smoked per day:<br>G1= 15 (8-30).<br>G2= 17 (9-32).<br>Time free from smoking among ex-smokers (years): G1= 4 (1-14).<br>G2= 3 (1-12).	G1= 85 (36,5%)<br>G2= 20 (26,3%)	Coronary arterial disease (CAD).	Coronary artery.
Sen S. et al. 2023	USA	Total= 28,2±5,0.<br>G1= 28,6±6<br>G2= 8,0±5,1	Total= 29,3 %<br>G1= 265 (77%).<br>G2= 559 (70%).	Never smoked= Total= 52,5%.<br>G1 = 189 (55%)<br>G2= 438 (55%).<br>Smokes= Total= 39,4%.<br>G1= 169 (49%)<br>G2= 400 (50%)<br>Smoked= Total= 8%.<br>G1 = 14 (4%).<br>G2= 27 (3%)	G1= 90 (56%)<br>G2= 223 (28%)	Asymptomatic intracranial atherosclerosis (ICAS).	Intracranial internal carotid artery, middle cerebral artery, anterior cerebral artery, posterior cerebral artery, basilar artery and vertebral artery.

Supplementary Table 3. Characteristics of the population

Shen et al. 2023	China	BMI (kg/m2)= G1= 24,27 G2= 23,71.	N/D	Never smoked= G1: 38%, G2: 67%. Smokes= G1: 52%, G2: 30% Smoked= G1: 10%, G2: 3%	Duration of diabetes (Years): G1 = 14,2 ± 8,27 G2= 6,6 ± 6,68	Coronary atherosclerosis	Coronary artery
Ahn et al. 2016	Korea	N/D	G1 (Subclinical Atherosclerosis) with hypertension: 204 (55,4%) G2 (Non-Subclinical Atherosclerosis) with hypertension: 417 (42,8%)<br>G1 (peripheral arterial disease) with hypertension: 38 (52,8%) G2 (No peripheral arterial disease) with hypertension: 38 (52,8%)	Subclinical atherosclerosis At some point in life= G1: 45 (12,2). G2: 116 (11,9) Never= G1: 323 (87,8). G2: 859 (88,1)<br>PAD At some point in life= G1:12 (16,7) G2: 149 (11,7) Never= G1: 60 (83,3) G2: 1122 (88,3)	Total diabetics = 204 Total non-diabetics = 1139 Subclinical aterosclerosis = G1: Diabetes= 76 (20,7%) No diabetes= 292 (79,3%) G2= Diabetes= 128 (13,1%). No diabetes= 847 (86,9%)<br>PAD= G1: Diabetes= 13 (18,1%) No diabetes= 59 (81,9%) G2: Diabetes= 191 (15%) No diabetes= 1080 (85%)	Subclinical atherosclerosis and Peripheral arterial disease.	cIMT = Left and right common carotid artery. ABI = Upper and lower extremities
Thayana S et al. 2020	Brazil	BMI (> 25): G1= 38 (51,4%) G2= 209 (62%)	Hypertension: G1: 258 (75%) G2: 66 (89,2%)	Smokers and ex-smokers G1= 33 (44,6) G2= 120 (34,9)	G1= 19 (25,7%) G2= 63 (18,3%)	Carotid atherosclerotic burden (CAB)	Common, internal and external carotid arteries.
H.yu 2014	China	G1= 26,40 ± 3,33 G2= 25,59 ± 3,91	G1= Yes: 137 (37,0) No: 108 (22,6) G2= Yes: 233 (63,0) No: 369 (77,4)	Smoking status: G1: Yes= 107 (37,3) No= 138 (24,6) G2: Yes = 180 (62,7) No = 422 (75,4)	Blood glucose (fasting): G1= 6,79 ± 2,47 G2= 6,20 ± 2,16	Subclinical atherosclerosis	Common carotid artery

Supplementary Table 3. Characteristics of the population

.

Shimizu, Y, et al. 2022	Japan	G1= 23,2 ± 3,0<br>G2= 22,9 ± 3,5	G1= (systolic) SBP= 144 ± 19, (diastolic) DBP= 83 ± 12<br>G2= SBP= 135 ± 19, DBP= 82 ± 11	Current smokers=<br>G1= 6,9%<br>G2=8,7%	Glycosylated hemoglobin:<br>G1= 5,9 ± 0,6<br>G2= 5,7 ± 0,5	Functional atherosclerosis.	Common carotid arteries
Ahmed J et al. 2022	India	N/D	N/D	N/D	N/D	Carotid artery calcification.	Internal carotid artery (ICA).
Lazzari de Onofre et al. 2021	Brazil	N/D	Total= 75 (26.4%)<br>G1= 55 (30.7%)<br>G2= 20 (19%)	Total=<br>Yes: 199 (70,1%), No: 85 (29,9%)<br>G1= Yes: 122 (68,2%), No: 57 (31,8%)<br>G2= Yes: 77 (73,3%), No: 28 (26,7%).	Total = Diabetics: 17 (6 %) No diabetes: 268 (94%).<br>G1= Diabetics: 14 (7,8%). No diabetes: 165 (92,2%)<br>G2= Diabetics: 3 (2,9%). No diabetes: 102 (97,16%)	Carotid artery calcification.	Internal carotid artery (ICA).

CAC: Coronary Artery Calcification, cIMT: Carotid Intima Media Thickness, ABI: Ankle Brachial Index, SBP: systolic blood pressure, DBP: diastolic blood pressure, N/D: No Data.
