# Supplementary material for: Association between atherosclerosis and tooth loss in adult patients: systematic review and meta-analysis
Source: Evid Based Dent. 2026 Mar 18;27(2):42–3. doi: 10.1038/s41432-026-01215-1 (PMC13309286; doi:10.1038/s41432-026-01215-1)
Supplement: Supplementary file 4 — Supplementary Table 4. Certainty of evidence. [file 41432_2026_1215_MOESM4_ESM.pdf]

**Supplementary Table 4.** Certainty of evidence. Individuals exposed to atherosclerosis versus those not exposed to determine the association with tooth loss

| Certainty assessment | | | | | | | № of patients | | Effect | | Certainty | Importance |
|---|---|---|---|---|---|---|---|---|---|---|---|---|
| **№ of studies** | **Study design** | **Risk of bias** | **Inconsistency** | **Indirectness** | **Imprecision** | **Other considerations** | **participants exposed to atherosclerosis** | **those not exposed to atherosclerosis** | **Relative (95% CI)** | **Absolute (95% CI)** | | |
| **Missing teeth** | | | | | | | | | | | | |
| 8[1,2,3,4,5,6,7,8] | non-randomised studies | serious[a] | serious[b] | serious[c] | not serious[d] | none | 35104/102368 (34.3%) | 67264/102368 (65.7%) | **MD 3.08** (1.80 to 4.37) | **-- per --** (from -- to --)[e] | ⊕◯◯◯ Very low[a,b,c,d] | IMPORTANT |
| **Severe tooth loss (≥20 teeth)** | | | | | | | | | | | | |
| 4[9,10,11,12] | non-randomised studies | serious[f] | very serious[g] | serious[c] | serious[h] | none | 306/2488 (12.3%) | 2182/2488 (87.7%) | **OR 1.27** (0.67 to 2.40) | **24 more per 1.000** (from 50 fewer to 68 more) | ⊕◯◯◯ Very low[c,f,g,h] | IMPORTANT |

**CI:** confidence interval; **OR:** odds ratio

**Explanations**

a. Two out of eight studies had a high risk, and three out of eight studies had an unclear risk.

b. There is high statistical heterogeneity ($I^2 = 99\%$) and several confounding factors. Additionally, the second subgroup has a wide confidence interval. Therefore, we consider the inconsistency to be serious.

c. Although the included studies assessed the same population and exposure defined in the PICO question, in several studies, their outcome of interest was not the primary objective; however, the studies reported it as a secondary finding. Therefore, we rated indirectness as serious.

d. The pooled estimate showed a narrow 95% confidence interval (MD = 3.08, 95% CI 1.80–4.37), not crossing the line of no effect. The sample size was adequate; therefore, imprecision was not considered serious.

e. Outcome is continuous (difference of means). The effect absolute corresponds to the difference in scale units.

f. Two out of four studies had an unclear risk of bias.

g. Heterogeneity among studies was high. Two studies show no effect, and the other two studies have effects in opposite directions. We rated inconsistency very serious (downgraded two levels).

h. Two of the four studies cross the line of no effect, although confidence intervals are not wide. We rated imprecision serious.

**References**


1.Donders H, IJzerman L,Soffner M,van't Hof A,Loos B,de Lange J. Elevated Coronary Artery Calcium scores are associated with tooth loss. PLOS ONE.; 2020.

2.Donders H, Veth E,van 't Hof A,de Lange J,Loos B. The association between periodontitis and cardiovascular risks in asymptomatic healthy patients. Int J Cardiol Cardiovasc RISK Prev.; 2021.

3.Yu H, Qi LT,Liu LS,Wang XY,Zhang Y,Huo Y,et al. Association of Carotid Intima–media Thickness and Atherosclerotic Plaque with Periodontal Status. J Dent Res.; 2014.

4.Ahn YB, Shin MS,Han DH,Sukhbaatar M,Kim MS,Shin HS,et al. Periodontitis is associated with the risk of subclinical atherosclerosis and peripheral arterial disease in Korean adults. Atherosclerosis.; 2016.

5.Shen M, Li Z,Li H,Yan X,Feng B,Xu L. Association of periodontitis and tooth loss with extent of coronary atherosclerosis in patients with type 2 diabetes mellitus. Front Endocrinol.; 2023.

6.Soto-Barreras U, Olvera-Rubio JO,Loyola-Rodriguez JP,Reyes-Macias JF,Martinez-Martinez RE,Patiño-Marin N,et al. Peripheral arterial disease associated with caries and periodontal disease. J Periodontol.; 2013.

7.de Onofre NML, Vizzotto MB,Wanzeler AMV,Tiecher PF da S,Arús NA,Arriola Guillén LE,et al. Association between internal carotid artery calcifications detected as incidental findings and clinical characteristics associated with atherosclerosis: A dental volumetric tomography study. .Eur J Radiol.; 2021.

8.Ahmed J, T N,Sujir N,Shenoy N. Missing Teeth as an Early Predictive "Sign" for Atherosclerosis: A Retrospective Study. ARYA Atheroscler J.; 2022.

9.Gomes M, Chagas P,Padilha D,Caramori P,Hugo F,Schwanke C,et al. Association between self-reported oral health, tooth loss and atherosclerotic burden. Braz ORAL Res.; 2012.

10.Sen S, Meyer J,Mascari R,Trivedi T,Suri F,Wasserman B,et al. Association of Dental Infections with Intracranial Atherosclerotic Stenosis. .Cerebrovasc Dis Basel Switz.; 2024.

11.Leao TS, Tomasi G,Ibrahim MS,Conzatti L,Marrone LP,Reynolds MA,et al. Tooth loss is associated with atherosclerosis and a poorer functional outcome among stroke patients. Clin Oral Investig.; 2020.

12.Shimizu Y, Yamanashi H,Kitamura M,Miyata J,Nonaka F,Nakamichi S,et al.. Tooth Loss and Carotid Intima-Media Thickness in Relation to Functional Atherosclerosis: A Cross-Sectional Study. .J Clin Med. ; 2022.
