# Supplementary figures and images for: Association between atherosclerosis and tooth loss in adult patients: systematic review and meta-analysis
Source: Evid Based Dent. 2026 Mar 18;27(2):42–3. doi: 10.1038/s41432-026-01215-1 (PMC13309286; doi:10.1038/s41432-026-01215-1)

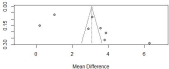

Supplement: Supplementary file 8 — Supplementary Fig. 1. Funnel plot Tooth loss in patients exposed and unexposed to atherosclerosis [file 41432_2026_1215_MOESM8_ESM.pdf]
