# Supplementary material for: Association between atherosclerosis and tooth loss in adult patients: systematic review and meta-analysis
Source: Evid Based Dent. 2026 Mar 18;27(2):42–3. doi: 10.1038/s41432-026-01215-1 (PMC13309286; doi:10.1038/s41432-026-01215-1)
Supplement: Supplementary file 10 — Supplementary Fig. 3. Forest Plot of tooth loss in patients exposed and unexposed to atherosclerosis according to the diagnostic tools. [file 41432_2026_1215_MOESM10_ESM.pdf]

Source	MD (95% CI)
**Subgroup2 = CT-scan**	
Shen 2023	3.57 [3.23; 3.91]
Donders 2020	2.90 [2.56; 3.24]
Donders 2021	0.20 [-0.10; 0.50]
Total	2.22 [-2.21; 6.66]
Heterogeneity: χ^2_2 = 253.25 (P < .001), I^2 = 99.2%	
**Subgroup2 = ABI**	
Soto-Barreras 2013	3.80 [3.25; 4.35]
**Subgroup2 = cIMT by ultrasound**	
Ahn 2016	3.10 [2.94; 3.26]
H Yu 2014	1.02 [0.89; 1.15]
Total	2.06 [-11.16; 15.27]
Heterogeneity: χ^2_1 = 378.02 (P < .001), I^2 = 99.7%	
**Subgroup2 = CBCT-scan**	
Ahmed 2022	6.29 [5.72; 6.86]
Lazzari de Onofre 2021	3.87 [3.46; 4.28]
Total	5.07 [-10.30; 20.45]
Heterogeneity: χ^2_1 = 45.78 (P < .001), I^2 = 97.8%	
Total	3.08 [1.53; 4.64]

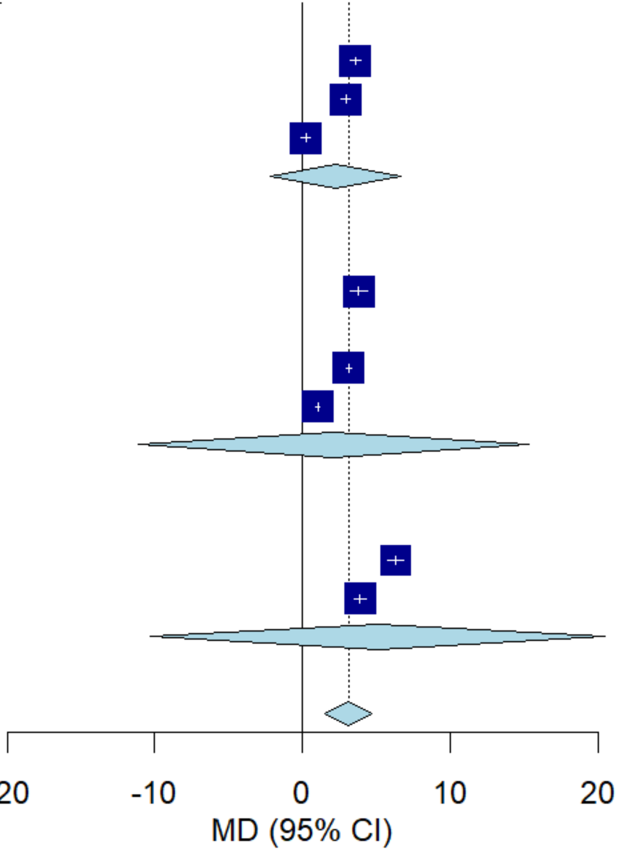

Heterogeneity: χ^2_7 = 970.25 (P < .001), I^2 = 99.3%
Test for subgroup differences: χ^2_3 = 5.92 (P = .12)
