# Supplementary material for: Association between atherosclerosis and tooth loss in adult patients: systematic review and meta-analysis
Source: Evid Based Dent. 2026 Mar 18;27(2):42–3. doi: 10.1038/s41432-026-01215-1 (PMC13309286; doi:10.1038/s41432-026-01215-1)
Supplement: Supplementary file 11 — Supplementary Fig. 4. Forest Plot of severe tooth loss in patients exposed and unexposed to atherosclerosis according to the diagnostic tools. [file 41432_2026_1215_MOESM11_ESM.pdf]

Source	OR (95% CI)
**Subgroup = Angiography**	
Schünke Gomes, 2012	0.56 [0.36; 0.87]
Sen, 2023	1.31 [0.91; 1.87]
Thayana, 2020	2.81 [1.74; 4.55]
Total	1.27 [0.17; 9.28]
Heterogeneity: $\chi^2_2 = 23.7$ ($P < .001$), $I^2 = 91.6\%$	
**Subgroup = cIMT by ultrasound**	
Shimizu, 2022	1.29 [0.96; 1.73]
Total	1.27 [0.45; 3.56]

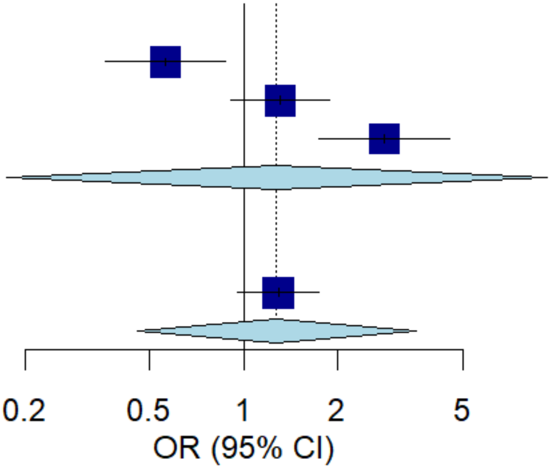

Heterogeneity: $\chi^2_3 = 23.76$ ($P < .001$), $I^2 = 87.4\%$

Test for subgroup differences: $\chi^2_1 = 0.00$ ($P = .98$)
