# Supplementary material for: Association between atherosclerosis and tooth loss in adult patients: systematic review and meta-analysis
Source: Evid Based Dent. 2026 Mar 18;27(2):42–3. doi: 10.1038/s41432-026-01215-1 (PMC13309286; doi:10.1038/s41432-026-01215-1)
Supplement: Supplementary file 12 — Supplementary information [file 41432_2026_1215_MOESM12_ESM.docx]

# List of Supplementary Information:

Supplementary Table 1. Search Strategy. Types of files PDF Supplementary Table 2. Citations excluded. Types of files PDF

Supplementary Table 3. Characteristics of the population. Types of files PDF

Supplementary Table 4. Certainty of evidence. Individuals exposed to atherosclerosis versus those unexposed to determine the association with tooth loss.

Supplementary Table 5. Meta-analyses Of Observational Studies in Epidemiology (Moose) Checklist

Supplementary Table 6. Preferred Reporting Items for Systematic Reviews and Meta-analysis (PRISMA) 2020 for Abstracts Checklist

Supplementary Table 7. Preferred Reporting Items for Systematic Reviews and Meta-analysis (PRISMA) 2020 Checklist

Supplementary Figure 1. Funnel plot Tooth loss in patients exposed and unexposed to atherosclerosis. Types of files PDF

Supplementary Figure 2. Risk of bias assessment Case control. Types of files PDF

Supplementary Figure 3. Forest Plot of tooth loss in patients exposed and unexposed to atherosclerosis according to the diagnostic tools. Types of files PDF

Supplementary Figure 4. Forest Plot of severe tooth loss in patients exposed and unexposed to atherosclerosis according to the diagnostic tools. Types of files PDF
