# Supplementary material for: Association between atherosclerosis and tooth loss in adult patients: systematic review and meta-analysis
Source: Evid Based Dent. 2026 Mar 18;27(2):42–3. doi: 10.1038/s41432-026-01215-1 (PMC13309286; doi:10.1038/s41432-026-01215-1)
Supplement: Supplementary file 9 — Supplementary Fig. 2. Risk of bias assessment Case control [file 41432_2026_1215_MOESM9_ESM.pdf]

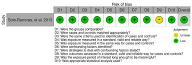
Risk of bias
D1
D2
D3
D4
D5
D6
D7
D8
D9
D10
Overall
Study
Soto-Barreras, et al. 2013
+
-
D1: Were the groups comparable?
D2: Were cases and controls matched appropriately?
D3: Were the same criteria used for identification of cases and controls?
D4: Was exposure measured in a standard, valid and reliable way?
D5: Was exposure measured in the same way for cases and controls?
D6: Were confounding factors identified?
D7: Were strategies to deal with confounding factors stated?
D8: Were outcomes assessed in a standard, valid and reliable way for cases and controls?
D9: Was the exposure period of interest long enough to be meaningful?
D10: Was appropriate statistical analysis used?
Judgement
Unclear
Low
